# Clinical effectiveness and safety of intraoperative methadone in patients undergoing laparoscopic hysterectomy: a randomised, blinded clinical trial

**DOI:** 10.1016/j.bjao.2023.100219

**Published:** 2023-08-05

**Authors:** Kristian D. Friesgaard, Lone D. Brix, Christina B. Kristensen, Omar Rian, Lone Nikolajsen

**Affiliations:** 1 Department of Anaesthesiology and Intensive Care, Horsens Regional Hospital, Horsens, Denmark; 2 Department of Anaesthesiology and Intensive Care, Aarhus University Hospital, Aarhus, Denmark; 3 Department of Gynaecology, Horsens Regional Hospital, Horsens, Denmark

**Keywords:** acute postoperative pain, laparoscopy, methadone, pain management, postoperative

## Abstract

**Background:**

Laparoscopic hysterectomy is often carried out as day-stay surgery. Minimising postoperative pain is therefore of utmost importance to ensure timely discharge from hospital. Methadone has several desirable pharmacological features, including a long elimination half-life. Therefore, a single intraoperative dose could provide long-lasting pain relief.

**Methods:**

Patients scheduled to undergo laparoscopic hysterectomy were randomly allocated to receive methadone (0.2 mg kg^−1^) or morphine (0.2 mg kg^−1^) intraoperatively, 60 min before tracheal extubation. Primary outcomes were opioid consumption (oral morphine equivalents in milligrams) at 6 and 24 h. Secondary outcomes included pain intensity at rest and during coughing, patient satisfaction, postoperative nausea and vomiting, and adverse events up to 72 h after completion of surgery.

**Results:**

The postoperative median opioid consumption was reduced in the methadone group compared with the morphine group at 6 h (35.5 [0–61] mg *vs* 48 [31–74.5] mg; *P*=0.01) and 24 h (42 [10–67] mg *vs* 54.5 [31–83] mg; *P*=0.03). On arrival at the PACU, pain at rest was significantly lower in patients receiving methadone (numeric rating scale: 3 [2–5] *vs* 5 [3–6]), whereas pain scores at rest and coughing were not significantly different throughout the rest of the observation period. No differences in other secondary outcomes were found.

**Conclusions:**

In this randomised, blinded, controlled trial, opioid consumption was reduced during the first 24 postoperative hours in patients receiving methadone without causing an increase in adverse events. The difference observed might be considered as small and of limited clinical relevance.

**Clinical trial registration:**

NCT03908060; EudraCT no. 2018-004351-20.

Laparoscopic hysterectomy is a common gynaecological procedure and is increasingly being performed as a day-stay procedure to aid recovery and increase patient turnover.^1^ In day-stay surgery, shorter-acting anaesthetic and analgesic drugs used during the procedure enable rapid awakening and meeting of discharge criteria. Despite using a multimodal analgesic regimen with paracetamol, NSAIDs, and peripheral nerve blocks (if applicable),^2^ this approach may, however, be associated with rapidly waning serum opioid concentrations, thereby increasing the risk of inadequate pain control and unnecessary postoperative suffering. Consequently, many patients undergoing laparoscopic hysterectomy experience excessive pain and discomfort in the postoperative period.^3^^,^^4^

A balanced approach with a single intraoperative dose of a long-acting opioid that could extend the analgesic effect is therefore desirable. This could potentially reduce fluctuations in serum opioid concentrations and thereby the consumption of postoperative opioids. Methadone has several suitable pharmacological properties for the treatment of acute pain, including a long elimination half-life^5^^,^^6^ and multi-target effect on nociceptors in the CNS.^7^^,^^8^ Methadone has traditionally proved efficacious in replacement strategies for addicts^9^ and in chronic pain conditions.^10^, ^11^, ^12^ Some studies have tested methadone as a treatment for acute pain in the operating theatre or PACU, many of which have revealed opioid-sparing effects and significant pain relief in paediatric,^13^ open heart,^14^^,^^15^ spinal fusion,^16^^,^^17^ and general surgery.^6^ The role in relieving acute postoperative pain in laparoscopic procedures remains largely unexplored.^18^, ^19^, ^20^

The objective of this study was to examine whether a single dose of intraoperative methadone will reduce postoperative opioid consumption and pain after laparoscopic hysterectomy compared with standard care, which includes morphine. Secondarily, the aim was to assess the occurrence of opioid-related side-effects in the two treatment arms.

## Methods

### Study design and study population

This study was a single-centre, investigator-initiated, prospective, randomised, blinded trial using two parallel groups. The study was conducted in accordance with the Declaration of Helsinki and Guidelines for Good Clinical Practice (GCP) and monitored by the GCP unit at Aarhus University Hospital, Aarhus, Denmark. The study protocol was approved by the Danish Protection Agency (ID 1-16-02-747-18), the Central Denmark Region Committees on Health Research Ethics (ID 1-10-72-365-18), and the Danish Health and Medicines Authority (ID 2018-004351-20). The study was registered (9 April 2019) at ClinicalTrials.gov (NCT03908060) and EudraCT no. 2018-004351-20, and the protocol was published at the initial stage of the trial.^21^ All authorities approved a change of sample size in May 2021, and ClinicalTrials.gov was updated accordingly.

Patients were screened for inclusion at the first ambulatory contact at Horsens Regional Hospital, Horsens, Denmark, and informed oral and written consent was obtained before surgery. Adult females scheduled for day-stay, elective, laparoscopic hysterectomy were enrolled with the following exclusion criteria: ASA physical status 4 or 5,^22^ prolonged QT interval assessed by electrocardiogram (>440 ms), existing treatment with medications prolonging the QT interval, hysterectomy for malignancy or acute bleeding disorders, allergy to study drugs, preoperative daily use of opioids, inability to provide informed consent, and intraoperative conversion to open surgery.

### Randomisation and blinding

Included patients were randomly assigned in a 1:1 ratio to receive either intraoperative i.v. methadone (Streuli Pharma AG, Uznach, Switzerland) or morphine (Amgros I/S, Copenhagen, Denmark), using a computer-generated block randomisation procedure with blocks of varying sizes (12–20). Randomisation and study drugs were managed by the pharmacy at Aarhus University Hospital, and the randomisation list was concealed until completion of all statistical analyses. The study drugs were prepared in identical, colourless, sequentially numbered 10 ml syringes and contained a concentration of methadone or morphine corresponding to 2 mg ml^−1^. Each syringe was marked with batch, randomisation, and the patient's personal registration number and delivered to the investigators on the day of surgery. All investigators, healthcare professionals, and patients were in this way blinded to the treatment allocation.

### Intraoperative management

On the morning of surgery, the included patients received paracetamol 1000 mg and ibuprofen 400 mg. Following anaesthetic induction, all patients received dexamethasone 8 mg intravenously.^23^ Anaesthesia was induced and maintained using a standard protocol with propofol and remifentanil (doses adjusted according to bispectral index values^24^ and clinical judgement of anaesthesia depth), and standard monitoring of non-invasive BP, continuous cardiac monitoring (electrocardiogram), and peripheral oxygen saturation was applied. Vasopressors, neuromuscular blocking agents, and i.v. crystalloids were administered at the discretion of the anaesthesiologist. Truncal regional nerve blocks were not performed. Surgery was performed laparoscopically (maximum permitted insufflation pressure was 12 mm Hg) following international standards.^25^ In some cases, a large uterus was extracted through a minor laparotomy. Infiltration of bupivacaine 2.5% (5 ml) into the skin and fascia before incision and injection of ropivacaine 2% (50 ml) intraperitoneally before closure was also standard. The study drugs were administered in equipotent doses (0.2 mg kg^−1^) equivalent to 1 ml for every 10 kg of ideal body weight (height [cm]–105) approximately 60 min before tracheal extubation. Tracheal extubation took place in the operating theatre before the patient was transferred to the PACU for further treatment and observation until hospital discharge.

### Postoperative management

Pain intensity was assessed on arrival in the PACU arrival and every 15 min thereafter using a numeric rating scale (NRS; 0–10, where 0=no pain and 10=worst possible pain). Patients were specifically assessed for abdominal pain rather than pain elsewhere (e.g. shoulder pain related to laparoscopy and abdominal insufflation). PACU nurses applied a standard treatment protocol for the treatment of postoperative pain. Moderate pain (NRS >3) was treated with i.v. morphine (initial dose 0.1 mg kg^−1^ [<75 yr] and 0.05 mg kg^−1^ [>75 yr]) and titrated (50% of initial dose) every 10 min until NRS ≤3. Supplementary oral morphine was administered (10 mg [<75 yr] or 5 mg [>75 yr]) when necessary. If there was an inadequate response to morphine, i.v. oxycodone and oral oxycodone were administered following the same procedure and dose as morphine. In case of severe pain intensity (NRS >6), morphine was supplemented with i.v. fentanyl (single dose of i.v. fentanyl 50 μg [adults between 18 and 75 yr] or i.v. fentanyl 25 μg [adults <75 yr, weight <50 kg, or kidney disease]). Opioid analgesia was supplemented with oral paracetamol and ibuprofen. Dexamethasone, ondansetron, droperidol, and cyclizine were used for prophylaxis and treatment of postoperative nausea and vomiting (PONV).^26^ Recording of the administration of all medicines in an electronic hospital record was mandatory. Most patients were discharged directly from PACU on the day of surgery unless there were complications or postoperative symptoms persisted. After hospital discharge, pain treatment consisted of paracetamol (1000 mg × 4 per day), ibuprofen (400 mg × 3 per day), and oral morphine 10 mg if indicated (10 pills prescribed for each patient).

### Outcomes

The primary outcomes were the accumulated opioid consumption in the first 6 and 24 postoperative hours reported as oral morphine equivalents.^27^ Secondary outcomes were pain intensity (NRS 0–10) at rest and coughing at 1, 6, 24, and 48 h; patient satisfaction with pain management at 3 and 24 h (NRS 0–10); PONV (none/mild/moderate/severe) at 6, 24, and 72 h; time from completion of surgery to discharge from the PACU; level of sedation (Ramsay Sedation Scale^28^ Level 2; awake and cooperative, oriented, or tranquil) at 0.5, 1, and 3 h; and adverse events during observation in the PACU (hypoventilation: ventilatory frequency <10 min^−1^; hypoxaemia: peripheral oxygen saturation <94%). The following patient characteristics and procedural data were recorded: age, BMI, ASA score, Charlson Comorbidity Index, tobacco and alcohol use, education, previous history of abdominal surgery/Caesarean section/number of births/menopause, relevant time points (start and end of anaesthesia and surgery), study medication administration and tracheal extubation, doses of propofol and remifentanil, fluids administered, blood loss, size of uterus, use of vasopressors or inotropes, and pain intensity in the entire follow-up period of 72 h. Data on opioid administration were obtained through hospital records, whereas all other data were obtained by direct examination of the patient or by telephone interview following discharge from hospital.

### Sample size estimation and statistical analysis

Calculation of sample size was based on a pre-trial audit of 70 patients scheduled to undergo hysterectomy at our department, revealing an overall opioid consumption (converted to oral morphine equivalent) of 50.5 mg before hospital discharge. The initial sample size plan (*n*=250) was redefined because of the COVID-19 pandemic and the massive cancellation and postponement of elective surgery in Denmark for several periods during the trial period. Hence, a total of 126 patients had to be included to find a 40% decrease in postoperative opioid use amongst patients allocated to methadone (80% power and an alpha level of 5%). Study data were collected and managed using the REDCap electronic data capture tools hosted at Aarhus University, Denmark.^29^ PACU nurses recorded patient and outcome data during the hospital stay, whereas the authors collected data by telephone interviews once the patients were discharged from PACU. Statistical analyses were performed using Stata software version 15.0 (StataCorp, College Station, TX, USA). Categorical data (number of patients with moderate or severe PONV, number of patients with Ramsay Sedation Scale Level 2, and any adverse events during observation at the PACU) are reported as numbers (%) with 95% confidence intervals and compared using the χ^2^ test. Medians with inter-quartile ranges are given for continuous skewed data (pain intensity, time from arrival to readiness for discharge from PACU, and patient satisfaction) and compared with the Mann–Whitney test. Data on postoperative opioid consumption did not follow a normal distribution and were compared with non-parametric tests as well. All *P*-values are two-sided, and those below 0.05 are considered significant.

## Results

**Figure 1:**
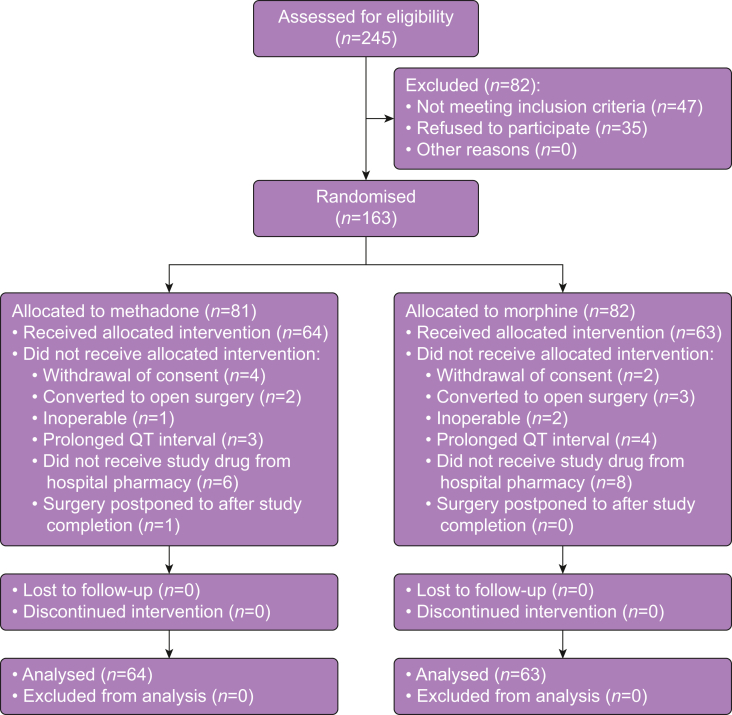
Participant flow diagram.

The results are reported according to the Consolidated Standards of Reporting Trials (CONSORT) statement.^30^ The trial period (May 2019–June 2022) was extended 1.5 yr because of an unexpected slow inclusion rate during the COVID-19 period. Patient flow is presented in Fig 1 with 163 patients randomly allocated to receive either methadone or morphine and 127 patients included in the final analyses. Baseline patient characteristics are provided in Table 1.

**Table 1:** Patient characteristics and perioperative data. Data presented as means with 95 confidence intervals (95% CIs) or medians with inter-quartile ranges (IQRs).

Group	Methadone (*n*=64)	Morphine (*n*=63)
Age (yr) (95% CI)	47.4 (45.7–49.1)	46.6 (44.6–48.6)
ASA physical status, *n* (%)		
1	29 (45.3)	26 (41.2)
2	31 (48.4)	36 (57.2)
3	3 (4.7)	1 (1.6)
Unknown	1 (1.6)	0 (0.0)
BMI (95% CI)	26.9 (25.7–28.0)	27.1 (26.3–28.7)
Minor laparotomy performed, *n* (%)	11 (17.2)	11 (17.5)
Bleeding (ml) (IQR)	50 (20–100)	75 (20–150)
Uterus weight (mg) (IQR)	216 (130–428)	204 (100–401)
Crystalloids (ml) (IQR)	1200 (1037–1400)	1250 (1100–1400)
Propofol 10 mg ml^−1^ (ml) (95% CI)	103.8 (95.5–112.1)	110.9 (102.5–119.4)
Remifentanil 50 μg ml^−1^ (ml) (95% CI)	87.2 (78.7–95.7)	93.7 (85.9–101.5)
Vasopressor (ephedrine), *n* (%)	53 (82.8)	48 (76.2)
Anaesthesia duration (min) (IQR)	160 (144–185)	160 (145–190)
Surgery duration (min) (IQR)	108 (90–130)	107 (91–120)
Time from study drug administration to extubation (min) (IQR)	65 (55–75)	63 (51–77)

The postoperative median oral opioid consumption was significantly reduced in patients treated with methadone compared with patients treated with morphine at 6 h (35.5 [0–61] mg *vs* 48 [31–74.5] mg; *P*=0.01) and 24 h (42 [10–67] mg *vs* 54.5 [31–83] mg; *P*=0.03). A *post hoc* analysis showed that the number of patients using no opioids in the first 6 postoperative hours was significantly higher in patients treated with methadone (18/64) compared with patients treated with morphine (7/63); *P*=0.01. This difference did not reach statistical significance after 24 postoperative hours (methadone: 10/64 *vs* morphine: 7/63; *P*=0.46).

**Figure 2:**
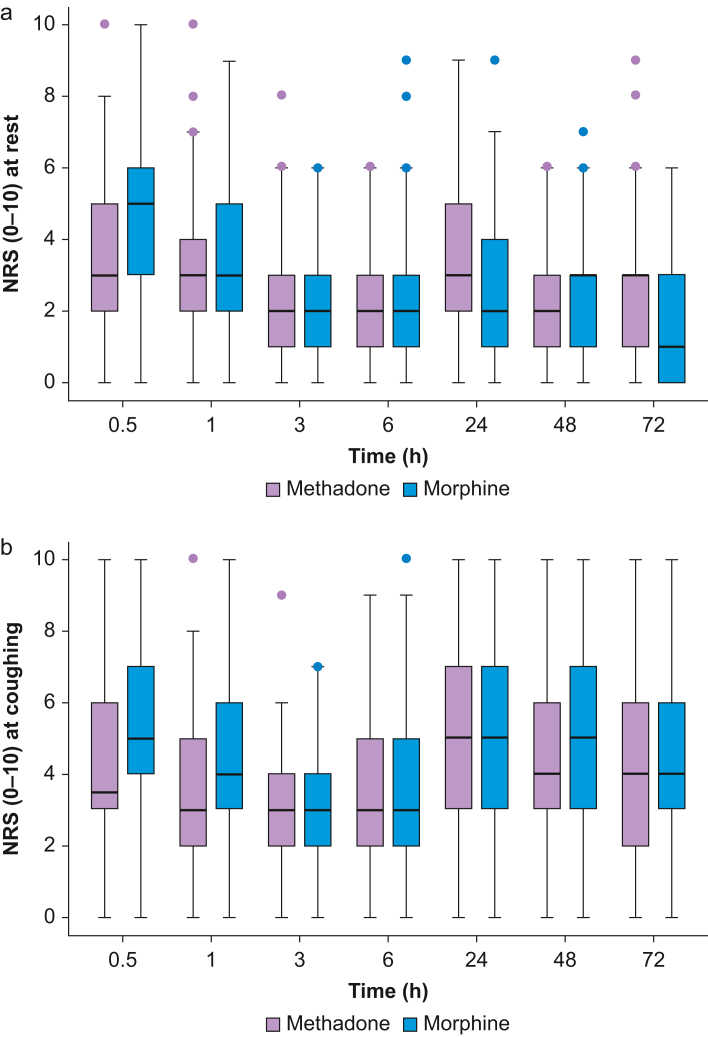
(a) Pain intensity at rest. (b) Pain intensity when coughing. NRS, numeric rating scale. The median is represented by the bold horizontal line within the box. The box defines the interquartile range (IQR) with the upper and lower whiskers defines values lying within 1.5 IQR of the upper and lower quartile respectively. Outliers are individual data points beyond 1.5 IQR of the upper quartile.

On arrival in the PACU, pain at rest was significantly lower amongst patients who had received methadone (3 [2–5] *vs* 5 [3–6]; *P*=0.01), whereas pain scores at rest and coughing were similar throughout the remaining observation period (Fig 2a and b). No differences in patient satisfaction, PONV, adverse events, or time until hospital discharge were found (Table 2). Furthermore, the proportion of patients discharged on the day of surgery was similar in both groups (*P*=0.65).

**Table 2:** Postoperative measures. Data presented as medians with inter-quartile ranges (IQRs), numbers, or proportions. ∗Awake and cooperative, oriented, and tranquil. NRS, numeric rating scale; PONV, postoperative nausea and vomiting.

Group	Methadone (*n*=64)	Morphine (*n*=63)
Same-day discharge, *n* (%)	47 (73.4)	44 (69.8)
Reason for overnight stay, *n*		
Pain	2	3
PONV	7	7
No reason specified	2	2
Other	6	7
Time in hospital (min) (IQR)	354 (273–998)	394 (318–1139)
Patient satisfaction, NRS (0–10)		
3 h	10 (9–10)	10 (9–10)
24 h	9 (7–10)	9 (8–10)
PONV at 6 h, *n* (%)		
None or mild	49 (76.6)	42 (67.7)
Moderate or severe	15 (23.4)	20 (32.3)
PONV at 24 h, *n* (%)		
None or mild	49 (76.6)	51 (81.0)
Moderate or severe	15 (23.4)	12 (19.0)
PONV at 72 h, *n* (%)		
None or mild	56 (88.9)	59 (93.7)
Moderate or severe	7 (11.1)	4 (6.3)
Ramsay Sedation Scale Level 2,∗ *n* (%) at		
0.5 h	50 (78.1)	47 (75.8)
1 h	44 (69.8)	45 (72.6)
3 h	52 (86.7)	57 (91.9)
Adverse events in PACU, *n*		
Ventilatory frequency <10 min^−1^	1	1
Oxygen saturation <94%	0	1

## Discussion

In this randomised, blinded, controlled trial, a single dose of methadone reduced opioid consumption during the first 24 postoperative hours compared with morphine. However, the observed differences were small, and a difference of 12.5 mg in oral morphine equivalents 6 and 24 h after surgery completion may not be considered as clinically relevant. Methadone only reduced postoperative pain at PACU arrival compared with morphine, and the observed side-effects and the proportion of same-day discharge were similar. Patients were equally satisfied with pain treatment in the two groups.

Two recent systematic reviews have suggested a beneficial effect of i.v. methadone in terms of relieving acute postoperative pain. Machado and colleagues^31^ evaluated the results of 13 RCTs and reported statistically significant mean differences in pain scores at rest of 1.09 at 24 h, 1.47 at 48 h, and 1.02 at 72 h. On movement, the mean differences in pain scores were 2.48 at 24 h, 2.03 at 48 h, and 1.34 at 72 h, respectively.^31^ D'Souza and colleagues^32^ examined 10 RCTs and found that seven studies reported lower postoperative pain scores in the study period (24–72 h), but data were not pooled because of substantial differences in reported pain scales. Both reviews reported statistically significant differences in postoperative opioid requirements after 24 h, but the clinical magnitude was less obvious: oral morphine equivalents 15 mg^32^ and i.v. morphine equivalents 8.42 mg,^31^ respectively. Furthermore, the studies varied considerably in terms of study quality, outcomes, safety reporting, sample size (20–156 patients), surgical intervention, intervention arm (methadone dosing and time of administration), and control arm (large variation in type and dosing of opioid). Therefore, it can be argued whether pooling of estimates and overall conclusions are reliable.

The role of intraoperative methadone has only been investigated in three studies on patients scheduled for laparoscopic procedures. Simoni and colleagues^18^ included 126 patients scheduled for laparoscopic cholecystectomy or hiatus hernia repair to receive methadone (0.1 mg kg^−1^), clonidine (2 μg kg^−1^), or placebo (saline) 5 min before surgery. All patients received perioperative metamizole and ketoprofen, target-controlled infusion of propofol and remifentanil, and rescue analgesia with i.v. tramadol in the PACU as needed. Fewer patients (11 out of 42=26.2%) in the methadone group experienced pain in the PACU (VAS ≥3) compared with clonidine (21 out of 42=50.0%) and placebo (23 out of 42=54.8%). There were no relevant differences in the time until wakening, duration of surgery, or PONV in the PACU. The long-term effects of methadone and the need for rescue analgesia were not reported, and it can be argued that the comparisons with clonidine and placebo were not equipotent.

Moro and colleagues^20^ randomly allocated 70 patients planned for laparoscopic cholecystectomy to receive methadone (0.1 mg kg^−1^) or morphine (0.1 mg kg^−1^) after induction of anaesthesia. Maintenance was achieved with propofol and remifentanil, and multimodal analgesia consisted of dexamethasone, metamizole, and ketoprofen. Pain scores in the PACU and 24 h after surgery were similar in both groups, and no clinically relevant differences in rescue analgesia were found. More patients (45.2%) receiving morphine were sedated in the PACU compared with patients receiving methadone (9.7%), whereas PONV, time of PACU stay, incidence of hypoxaemia, and a wide range of questionnaire-based dimensions of recovery did not differ.

Although the analgesic-sparing effect of methadone in laparoscopic procedures may be limited, it might be beneficial for patients undergoing more extensive surgical procedures with higher postoperative opioid requirements. One of the most robust studies included 120 patients undergoing complex spine surgery.^16^ In the group receiving intraoperative methadone, i.v. hydromorphone consumption was 4.8 mg (i.v. morphine ≈24 mg) lower 24 h after surgery compared with the group that received intraoperative hydromorphone. More strikingly, pain scores at rest, coughing, and movement were significantly lower in almost all assessments throughout the study period of 72 postoperative hours. In the same period, overall satisfaction with pain management was higher in the methadone group, and no differences in relevant side-effects were observed. It can be questioned whether the drugs were used in equipotent doses (hydromorphone 2 mg *vs* methadone 0.2 mg kg^−1^) and whether the different timing of study drug administration (methadone at anaesthetic induction and hydromorphone after the end of surgery) could have influenced outcomes.

This study has limitations. First, we had to redefine our sample size because of the COVID-19 pandemic and the concomitant long periods of nationwide lockdowns of elective surgery. The initial sample-size calculation was conservative (*n*=250) and aimed to show a 30% reduction in opioid requirements in the group given methadone (90% power and an alpha level of 5%).^21^ As most studies on single-shot methadone have found a 40–50% reduction in postoperative opioid requirement, a pragmatic approach was applied, and the same reduction was assumed for our study. An alternative way to deal with this issue would have been to end the trial prematurely, but irrespective of approach, bias has potentially been introduced, and small actual differences on effect and safety might have been overlooked. Therefore, the results should be interpreted in the light of these precautions. Second, a dose–response study has not been performed on patients undergoing laparoscopic hysterectomy, and the dosing of methadone was based on existing literature; most studies administer 0.2 mg kg^−1^,^14^^,^^16^^,^^17^^,^^33^ whereas 0.1 mg kg^−1^,^18^, ^19^, ^20^^,^^34^ 0.15 mg kg^−1^,^19^^,^^35^ 0.3 mg kg^−1^,^36^ and fixed doses of 20 mg^15^,^37^, ^38^, ^39^ have been reported as well. It can be argued that a lower dose would be appropriate for less invasive procedures, such as laparoscopy. However, pharmacokinetic studies suggest that methadone doses less than 10 mg fail to provide a long analgesic effect because of rapid redistribution to fatty tissues, whereas higher doses exhibit a prolonged effect because of slower systemic elimination.^5^^,^^6^ Third, it can be argued that we should have chosen a primary patient-centred outcome, such as pain or satisfaction with treatment, as this might reflect the patient experience better than accumulated opioid consumption. However, opioid consumption is a standardised objective outcome that enables comparisons across studies, whereas pain and satisfaction on a scale can be misleading. Fourth, the use of two primary outcomes could potentially have increased the risk of a false-positive result and should be taken into account when interpreting the data. Fifth, a total of 163 patients were randomly allocated, but only 127 were included in the final analysis. The hospital pharmacy had to be notified 5 days before the day of planned surgery to randomise and prepare the study medication. This increased the risk of dropouts attributable to COVID-19-related cancellations, withdrawal of consent, etc. We believe that dropouts were random. Last, it was decided to administer the study drug 60 min before estimated tracheal extubation to ensure a fair comparison between methadone and morphine and to ensure a reasonable possibility for both drugs to have an effect on postoperative outcomes. This approach eliminated the theoretical pre-emptive analgesic effect of the study drug, which might have increased patients' postoperative pain.

In conclusion, during the first 72 postoperative hours, a single intraoperative dose of methadone produced only a small reduction in opioid consumption and in pain scores at PACU arrival compared with an equipotent dose of morphine, and these differences might be considered as clinically irrelevant. Side-effects, discharge rates, and patient satisfaction were similar in the compared groups such that methadone cannot be routinely recommended for laparoscopic hysterectomy.

## Authors’ contributions

Study design: KDF, LDB, LN.

Study supervision: LN.

Study effectuation at study site: CBK.

Patient inclusion and data acquisition effectuation at study site: OR.

Support for the daily conduct of the trail: LDB.

Data collection: KDF, LDB.

Data extraction: KDF.

Data analysis: LN.

Statistical analyses: KDF.

Drafting of paper: KDF, LDB, CBK, OR.

Revising of paper: LN.

Approval of final paper: LDB, CBK, OR, LN.

## Declarations of interest

The authors declare that they have no conflicts of interest.

## Funding

10.13039/501100009708The Novo Nordisk Foundation (NNF180C0052692).
